# Professional Commitment Among Graduating Nursing Students: A Cross‐Sectional Study

**DOI:** 10.1002/nop2.70442

**Published:** 2026-01-30

**Authors:** Li‐Hung Tsai, Jun‐Yu Fan, Sum‐Fu Chiang

**Affiliations:** ^1^ Department of Nursing Chang Gung University of Science and Technology Taoyuan Taiwan; ^2^ Department of Nursing & Graduate Institute of Nursing Chang Gung University of Science and Technology Taoyuan Taiwan; ^3^ Division of Nursing Chang Gung Memorial Hospital at Linkou Taoyuan Taiwan; ^4^ School of Traditional Chinese Medicine Chang Gung University Taoyuan Taiwan; ^5^ Division of Colon and Rectal Surgery Chang Gung Memorial Hospital at Linkou Taoyuan Taiwan

**Keywords:** avoidance behaviour, clinical competence, nursing

## Abstract

**Aim:**

This study aimed to explore the level of nursing professional commitment and its influencing factors among graduating nursing students in Taiwan.

**Design:**

This was a cross‐sectional study.

**Methods:**

This study was conducted with 359 graduating nursing students at a technological university in northern Taiwan. The data were collected using paper‐and‐pencil questionnaires. The Nursing Professional Commitment Scale (NPCS), Perceived Stress Scale (PSS), Coping Behaviour Inventory (CBI), and Clinical Competence Scale (CCS) were used. Multiple linear regression analysis was performed on the data. A *p* < 0.05 was considered statistically significant.

**Results:**

The professional commitment of graduating nursing students was at a moderate level. The results of the regression analysis showed that the most significant factor associated with nursing professional commitment was engaging in nursing in the future, followed by clinical competence, interest in nursing, and avoidance behaviour.

**Conclusions:**

Graduating nursing students demonstrate a moderate level of professional commitment that requires improvement. Therefore, it is imperative for nursing educators to comprehensively consider and understand the various factors influencing nursing students' professional commitment and implement targeted interventions. Recommended interventions include competency‐based instructional designs and the integration of professional values and emotional intelligence into the curriculum to strengthen students' core competencies, enhance adaptive coping skills, and reinforce beliefs in the professional roles.

**Patient or Public Contribution:**

No patient or public contribution.

## 1 Introduction

Professional commitment significantly contributes to the healthy, stable, and sustainable development of nursing professionals (Cheng et al. 2021). Nursing professional commitment describes an individual's positive attitudes and behaviours toward the nursing profession, a willingness to invest effort in their work, and a desire to continue their career as a nurse (Shi and Cleofas 2022). Increased professional commitment among nurses can lower turnover intention and enhance nursing professional competence, job satisfaction, and quality of nursing care (Zhao et al. 2022). Professional commitment is correlated with the retention of nursing students, professional development, and the nursing quality in their future work (Zhao 2023). Nursing students who demonstrate higher levels of professional commitment during their college years are expected to exhibit enhanced commitment once they become registered nurses after graduation (Chang et al. 2024). Enhanced professional commitment is crucial for alleviating the nursing workforce shortage (Hua et al. 2022). Therefore, it is necessary to understand the level of professional commitment among graduating nursing students and the influencing factors.

The clinical practicum for undergraduate nursing students is a crucial stage for nursing students to develop commitment to the profession (Zhang et al. 2023). It allows nursing students to integrate the knowledge and skills acquired in the classroom and facilitates their understanding of clinical practices and professional development (Anyango et al. 2024). During clinical practicum, nursing students often face stressors such as lack of competence, fear of mistakes, traumatic experiences, difficulties in patient relationships, and evaluation anxiety (Tsai et al. 2024), which may negatively impact their professional commitment. Indeed, a longitudinal study found that higher perceived stress during practicum predicted lower professional commitment afterward (Zhao et al. 2022). Another study suggested that a positive clinical learning environment strengthens nursing students' perceived clinical competence, which subsequently enhances their professional commitment (Ayaz‐Alkaya et al. 2018).

Coping consists of repeated ‘cognitive and behavioral’ efforts made by individuals to manage intense stressors, whether they originate internally and/or externally (Alshahrani et al. 2018). A systematic review found that nursing students typically employ a mix of coping strategies, utilising effective methods like problem‐solving and staying optimistic in parallel with less effective approaches such as avoidance and transference (Chaabane et al. 2021). Effective coping strategies can shield nursing students from depersonalization and aid in preventing reduced personal accomplishment. Ineffective coping strategies and high stress are significant factors leading to intention to quit nursing education prior to graduation (Ma et al. 2022). Therefore, nursing educators should be concerned and aware of whether students' coping behaviours during practicums impact their professional commitment to nursing.

Due to the complexity of health‐illness phenomena, experiential learning is vital for enhancing the practical skill and clinical competence development in nursing students. The clinical practicum is essential experiential learning, a form of learning by doing, which plays a crucial role in strengthening nursing students' clinical competence (Sun et al. 2021). Ten Hoeve et al. (2020) showed that insufficient competence is negatively associated with invoice nurses' professional commitment. Therefore, it is necessary to understand whether undergraduate nursing students' clinical competencies impact their professional commitment.

Nursing students may achieve good individual experiences and self‐image easily and are more willing to make efforts to pursue the nursing profession if they are interested in nursing and have positive feelings about being a nurse (Nesje 2016). A previous study showed that nursing students who were extremely interested in nursing and had good feelings about working as a nurse demonstrated the most substantial increase in professional commitment from pre‐ to post‐practicum (Zhao et al. 2022). The goal of all nursing programs is to train students to become competent nurses. After completing their studies, nursing students are willing to contribute to the nursing field (Tsai et al. 2024). However, when the frequency and duration of stressor exposure surpass students' ability to handle them, it can lead to premature burnout, heighten their intention to leave nursing education and their reluctance to work as nurses after graduation (Ma et al. 2022). The professional commitment of nursing students largely reflects their level of dedication and positive attitudes toward their chosen profession (Li et al. 2023). Their level of professional commitment contributes to career decision‐making (Wang et al. 2018). Therefore, whether students' willingness to engage in nursing in the future affects their professional commitment must be examined.

A strong inclination to leave the profession is common among the nursing workforce worldwide (Alsubhi et al. 2020). According to surveys, the global attrition rate of new nurses ranges from 24.5% to 70.0% (Kim and Shin 2020). In Taiwan, up to 40.0% of the new graduates left their job after three months of employment (Chen et al. 2020). Professional commitment is a key factor in attracting and retaining a nursing workforce. Among graduating nursing students, professional commitment will play a crucial role in alleviating the future nursing shortage, which is important for nursing stability (Cheng et al. 2021; Hua et al. 2022). Therefore, the professional commitment of graduating nursing students warrants considerable attention. Several studies have explored the level and factors influencing professional commitment among nursing students in all grades (Kong et al. 2016; Zhang et al. 2023; Zhao et al. 2022). However, only a few have specifically focused on the professional commitment of graduating nursing students. Relevant information regarding the professional commitment of graduating nursing students and its influencing factors may aid nurse educators in implementing effective interventions and facilitating the smooth transition from graduating nursing students to registered nurses.

### 1.1 Aims, Research Questions, and Objectives

#### 1.1.1 Aim

This study aimed to explore the level of professional commitment and its influencing factors among graduating nursing students in Taiwan.

#### 1.1.2 Research Questions

1. What is the level of professional commitment among graduating nursing students?
2. Which factors significantly predict professional commitment among graduating nursing students?

#### 1.1.3 Objectives

1. To assess the level of professional commitment among graduating nursing students.
2. To identify the significant predictors of professional commitment among this cohort.

### 1.2 Theoretical Framework

This study is guided by the Social Cognitive Career Theory (SCCT) developed by Lent et al. (1994). SCCT provides a comprehensive framework for understanding how individuals develop career interests and make career choices through the dynamic interplay of self‐efficacy, outcome expectations, and personal goals. In this study, the research variables are systematically mapped onto the SCCT model as significant predictors of professional commitment: *Self‐Efficacy (Clinical Competence)*: According to Lent et al. (1994), self‐efficacy refers to an individual's belief in their capability to organise and execute courses of action required to achieve specific attainments. In this context, clinical competence represents the self‐efficacy of nursing students. It is examined as a primary predictor reflecting the students' perceived mastery and confidence in their professional role. *Outcome Expectations (Perceived Stress and Coping Behaviour)*: Outcome expectations involve an individual's judgement of the likely consequences of their actions. Perceived stress during the clinical practicum represents the appraisal of environmental challenges, while coping behaviour reflects the regulatory mechanism used to manage these experiences. Together, these factors shape the students' expectations regarding the rewards and challenges of a nursing career. *Career Interests (Interest in Nursing)*: SCCT posits that career interests are formed in relation to one's perceived abilities and anticipated outcomes. In this study, interest in nursing is conceptualised as an intrinsic motivational driver that aligns students' personal preferences with their professional field. *Choice Goals (Engage in Nursing in the Future and Professional Commitment)*: Within the SCCT framework, ‘choice goals’ represent the intention to pursue, persist in, and excel within a specific career path. This study conceptualises the intention to engage in nursing in the future and the level of professional commitment (the dependent variable) as the ultimate choice goals.

By applying this framework, the study systematically examines how these independent variables (clinical competence, perceived stress, coping behaviour, interest in nursing, and engagement in nursing in the future) significantly predict the professional commitment of graduating nursing students in Taiwan.

## 2 Methods

### 2.1 Design, Setting and Participants

A cross‐sectional quantitative design was employed for this study. As noted by Cathala and Moorley (2018), a quantitative approach is ideal for examining the relationships between variables and illustrating these links objectively through statistical data. This methodology was selected to precisely determine the strength and direction of factors predicting professional commitment among graduating nursing students. Furthermore, convenience sampling was utilised to recruit fourth‐year students enrolled in the four‐year Bachelor of Science in Nursing (BSN) program at a technological university in northern Taiwan. This sampling method allowed for the recruitment of readily available participants who met the specific inclusion criteria prior to their graduation (Shorten and Moorley 2014). This approach ensured that data collection was systematic and objective, which is essential for ensuring the quality and repeatability of quantitative findings. The inclusion criteria targeted nursing students who were scheduled to graduate with a bachelor's degree in June 2022 and had completed their full‐time clinical practicum. Students who were suspended from school during the study period were excluded.

Out of 489 eligible fourth‐year nursing students, 360 provided written informed consent following an oral briefing and the distribution of a Participant Information Letter. The recruitment process emphasised the voluntary nature of the study, ensuring students could withdraw at any time without academic penalty. After excluding one incomplete response, the final sample consisted of 359 participants (Response rate: 73.4%). The 129 non‐participants were considered to have exercised their right to decline or withdraw from the study. The sample size was decided according to the rule of thumb, ensuring that a minimum of 10 participants was needed for each independent variable in the multiple regression analysis (Althubaiti 2023). In this study, the sample size had to be above 110 according to the number of independent variables, which were 11.

### 2.2 Measures

#### 2.2.1 Demographic Characteristics

Demographic characteristics variables, including age, gender (male, female), religion, high school attributes, interest in nursing, and engage in nursing in the future, were collected.

#### 2.2.2 Perceived Stress Scale (PSS)

The PSS, developed by Sheu et al. (1997), is a five‐point Likert scale designed to assess nursing students' stress and stressors. As the scale was originally developed and validated in Chinese within a Taiwanese nursing context, no translation process was required for this study. Responses range from ‘*never*’ to ‘*always*’ (0 = *never*, 1 = *infrequently*, 2 = *sometimes*, 3 = *frequently*, 4 = *always*). The scale divides into six subscales: eight for taking care of patients, six for teachers and nursing staff, five for assignments and workload, four for peers and daily life, three for lack of professional knowledge and skills, and three for the clinical environment. Mean scores for each item of the subscale and the overall scale were computed, with higher scores demonstrating higher stress levels. Regarding validity, Sheu et al. (1997) established content validity through an expert panel and used factor analysis to confirm the construct validity of the six subscales. In the previous study, Cronbach's *α* for the overall PSS was 0.89 (Sheu et al. 1997). In this study, the scale demonstrated excellent internal consistency with a Cronbach's *α* of 0.93.

#### 2.2.3 Coping Behaviour Inventory (CBI)

The CBI, developed by Sheu et al. (2001), is a five‐point Likert scale designed to assess nursing students' coping strategies. As the scale was originally developed and validated in Chinese within a Taiwanese nursing context, no translation process was required for this study. Responses range from ‘*never*’ to ‘*always*’ (0 = *never*, 1 = *infrequently*, 2 = *sometimes*, 3 = *frequently*, 4 = *always*). The inventory divides into four subscales: six related to avoidance behaviours, six related to problem‐solving behaviours, four related to stay optimistic behaviours, and three related to transference behaviours. Mean scores for each item in the subscale were computed, with higher scores demonstrating a higher frequency of adoption. Regarding validity, the original authors established content validity via an expert panel and confirmed construct validity using factor analysis. While the Cronbach's *α* for the CBI was reported as 0.76 (Sheu et al. 2001). In this study, Cronbach's *α* values were 0.82 for avoidance, 0.81 for problem‐solving, 0.77 for stay optimistic, 0.60 for transference, and 0.66 for overall. According to Portney and Watkins (2009), a Cronbach's *α* between 0.50 and 0.75 indicates moderate reliability, while values more than 0.75 indicate good reliability (Portney and Watkins 2009).

#### 2.2.4 Clinical Competence Scale (CCS)

The Clinical Competence Scale (CCS) was developed through a thorough literature review and clinical practice experience by the researcher. Through CCS, students self‐assessed their clinical competencies. The five‐point Likert scale was utilised in responses to 45 items ranging from ‘*extremely disagree*’ to ‘*extremely agree*’ (1 = *extremely disagree*, 2 = *disagree*, 3 = *neutral*, 4 = *agree*, 5 = *extremely agree*). The items were divided into five subscales: 27 related to general nursing, 5 related to cooperation, 4 related to management, 3 related to self‐growth, and 6 related to positivity (including stress adjustment, sense of responsibility, and service enthusiasm). Mean scores for each item in the subscale and overall scale were computed, with higher scores demonstrating greater clinical competence. Six nursing teachers and experienced nurses were invited to evaluate the scale's validity, scoring the questionnaire based on its universality, availability, definition and appropriateness. The results yielded a Content Validity Index (CVI) ranging from 0.97 to 1.0. Reliability was equally robust, with Cronbach's *α* values of 0.95 for the pilot testing and 0.96 for the main study.

#### 2.2.5 Nursing Professional Commitment Scale (NPCS)

The NPCS, developed by Lu and Chiou (1998), has been widely used to assess the professional commitment levels of nursing students and nurses. As the scale was originally developed and validated in Chinese within a Taiwanese nursing context, no translation process was required for this study. Responses to each item range from ‘*very unsure*’ to ‘*very sure*’ (1 = *very unsure*, 2 = *unsure*, 3 = *sure*, 4 = *very sure*). The scale divides into 4 subscales: 16 willingness to make an effort, which assesses how much effort one individual is willing to exert on behalf of the nursing profession; 8 desire to stay in the profession, reflecting a nursing student's intention to remain in the nursing profession; 5 intrinsic positive value of nursing work, reflecting the individual's perceived or calculated value of the nursing work; and 5 belief in goals and values, evaluating a student's belief in the goals and values of the nursing profession. Mean scores for each item in subscale and overall scale were computed, with higher scores demonstrating higher levels of professional commitment. Regarding validity, the original authors established content validity via an expert panel and confirmed construct validity using factor analysis. While the Cronbach's *α* for the NPCS was reported as 0.93 (Lu and Chiou 1998). In this study, the scale demonstrated excellent internal consistency with a Cronbach's *α* of 0.94.

### 2.3 Data Collection

Data for this study were collected in May 2022, prior to graduation. After obtaining ethics approval from the institutional review board, we explained the purpose and procedures of the study to the nursing students. To minimise social desirability bias and ensure data integrity, the survey was conducted anonymously under the supervision of a research assistant, self‐administered, pen‐and‐paper format in the classroom. Participation was entirely voluntary, and students were informed of their right to withdraw at any time without penalty. To further reduce potential bias, the course instructors were not present during the data collection process. After providing written informed consent, participants spent approximately 20 min completing the questionnaires. This structured approach ensured that students could respond honestly in a confidential environment.

### 2.4 Data Analysis

Data were analysed using SPSS 27.0. Reliability of the measurement tools was calculated using the Cronbach's *α* reliability test. Descriptive statistics were used to present participants' demographic characteristics, nursing professional commitment, perceived stress, coping behaviour, and clinical competence. Independent *t*‐test and one‐way ANOVA were used to compare the mean of professional commitment scores across demographic characteristics. The skewness and kurtosis values for the study variables were between −2 ~ 2, indicating that the data were normally distributed. Furthermore, Pearson correlation analysis was conducted to examine the relationships between continuous variables. The variance inflation factor (VIF) and tolerance were used to confirm multicollinearity. Multiple linear regression analysis was performed to analyse variables associated with nursing professional commitment. A *p* < 0.05 was considered statistically significant.

### 2.5 Ethical Considerations

This study was approved by the Institutional Review Board (201901913B0) of the institution where the study was conducted. The study adhered to the principles of research ethics. Prior to the study, the research process was explained to the nursing students and signed informed consent forms were obtained from them.

## 3 Results

### 3.1 Students' Demographic Characteristics and Their Relation to Nursing Professional Commitment

A total of 359 graduating nursing students participated in this study. The mean age of the participants was 21.40 (± 0.61) years; 316 (88.0%) were female; 210 (58.5%) had religious beliefs; 246 (68.5%) were senior high school graduates; 210 (58.5%) were interested in nursing, and 217 (60.5%) decided to engage in nursing in the future. The demographic characteristics of nursing students and their correlations with nursing professional commitment are shown in Table 1. To explore the factors affecting nursing professional commitment, univariate analysis was performed with demographic characteristics, of which only interested in nursing and engage in nursing in the future were significantly associated with nursing professional commitment.

**TABLE 1:** Students' demographic characteristics and univariate analysis related to nursing professional commitment (*N* = 359).

Variables	*n* (%)	M ± SD	Nursing professional commitment		
			M ± SD	*t/F*	*p*
Age		21.40 ± 0.61			
Gender^a^					
Male	43 (12.0)		2.97 ± 0.48	0.75	0.452
Female	316 (88.0)		2.90 ± 0.47		
Religious beliefs^a^					
Yes	210 (58.5)		2.93 ± 0.47	0.46	0.649
No	149 (41.5)		2.90 ± 0.47		
High school attributes^a^					
Senior high school	246 (68.5)		2.91 ± 0.49	−0.62	0.537
Vocational high school	113 (31.5)		2.94 ± 0.43		
Interested in nursing^b^					
Not interested at all	7 (1.9)		2.55 ± 0.59	17.83	< 0.001
Not interested in	19 (5.3)		2.63 ± 0.41		
Ordinary	123 (34.3)		2.76 ± 0.41		
Interested in	170 (47.4)		2.98 ± 0.45		
Very interested	40 (11.1)		3.34 ± 0.41		
Engage in nursing in the future^b^					
Very unsure	8 (2.2)		2.71 ± 0.68	56.29	< 0.001
Mostly unsure	18 (5.0)		2.16 ± 0.33		
A little unsure	45 (12.5)		2.55 ± 0.42		
Somewhat sure	71 (19.8)		2.72 ± 0.29		
Mostly sure	127 (35.4)		2.99 ± 0.31		
Very sure	90 (25.1)		3.32 ± 0.38		

Abbreviations: M, mean; SD, standard deviation.

^a^ Independent *t*‐test.

^b^ One‐way ANOVA.

### 3.2 Descriptive Statistics and Normality of Research Variables

The means, standard deviations, skewness, and kurtosis values of the variables are shown in Table 2. All the variables were normally distributed with skewness and kurtosis ranging from −1.0 to 1.0. The mean scores for each item of overall nursing professional commitment, overall perceived stress, avoidance behaviour, problem‐solving behaviour, stay optimistic behaviour, and transference behaviour were 2.92, 1.44, 1.02, 2.36, 2.58, and 2.02 out of 4 points, respectively. The mean score of each item of overall clinical competence and interest in nursing was 3.83 and 3.60 out of 5 points, respectively. The mean score for each item of engaging in nursing in the future was 4.56 out of six points.

**TABLE 2:** Means, standard deviations, skewness and kurtosis of variables (*N* = 359).

Variables	M ± SD	Skewness	Kurtosis
Nursing professional commitment	2.92 ± 0.48	−0.10	−0.01
Willingness to make an effort	2.70 ± 0.58		
Desire to stay in the profession	3.27 ± 0.49		
Intrinsic positive value of work	2.93 ± 0.55		
Belief in goals and values	3.04 ± 0.53		
Perceived stress	1.44 ± 0.49	0.36	0.72
Taking care of patients	1.64 ± 0.53		
Teachers and nursing staff	1.31 ± 0.57		
Assignments and workload	1.55 ± 0.73		
Peers and daily life	0.95 ± 0.67		
Lack of professional knowledge and skills	1.71 ± 0.61		
Clinical environment	1.35 ± 0.68		
Coping behaviour			
Avoidance	1.02 ± 0.67	0.51	0.09
Problem‐solving	2.36 ± 0.63	−0.15	0.76
Stay optimistic	2.58 ± 0.69	0.01	−0.33
Transference	2.02 ± 0.75	0.24	−0.10
Clinical competence	3.83 ± 0.51	−0.03	−0.04
General nursing	3.80 ± 0.53		
Cooperation	3.94 ± 0.61		
Management	3.89 ± 0.63		
Self‐growth	3.85 ± 0.65		
Positiveness	3.82 ± 0.69		
Interest in nursing	3.60 ± 0.83	−0.54	0.71
Engage in nursing in the future	4.56 ± 1.25	−0.82	0.12

Abbreviations: M, mean; SD, standard deviation.

### 3.3 Correlations Between Research Variables

Pearson correlation analysis showed that problem‐solving behaviour, stay optimistic behaviour, transference behaviour, clinical competence, interest in nursing, and engage in nursing in the future scores were positively correlated with nursing professional commitment scores. However, perceived stress and avoidance behaviour scores were negatively correlated with nursing professional commitment scores, as shown in Table 3.

**TABLE 3:** Correlation analysis (*N* = 359).

Variables	1	2	3	4	5	6	7	8	9
1. Nursing professional commitment	1								
2. Perceived stress	−0.35**	1							
3. Avoidance behaviour	−0.39**	0.65**	1						
4. Problem‐solving behaviour	0.27**	−0.17**	−0.16**	1					
5. Stay optimistic behaviour	0.39**	−0.48**	−0.53**	0.40**	1				
6. Transference behaviour	0.14*	−0.08	−0.08	0.31**	0.27**	1			
7. Clinical competence	0.40**	−0.48**	−0.39**	0.35**	0.42**	0.19**	1		
8. Interest in nursing	0.39**	−0.19**	−0.19**	0.08	0.20**	0.10	0.15**	1	
9. Engage in nursing in the future	0.33**	−0.19**	−0.19**	0.14*	0.15**	0.06	0.09	0.27**	1

* *p* < 0.05.

** *p* < 0.01.

### 3.4 Multiple Linear Regression Analysis on Nursing Professional Commitment of Nursing Students

A multiple linear regression analysis was conducted. The overall scores for nursing professional commitment were included as dependent variables, and perceived stress, avoidance behaviour, problem‐solving behaviour, stay optimistic behaviour, transference behaviour, clinical competence, interest in nursing, and engage in nursing in the future were included as independent variables. There was no multicollinearity among the independent variables. Variance analysis of the regression equation showed that the *F*‐value was 51.62 (*p* < 0.001), indicating that the fitted multiple linear regression equation was statistically significant. Results indicated that the eight variables explained 53.1% of the variance in nursing professional commitment (adjusted *R* ^2^ = 0.53). Of these variables, avoidance behaviour, clinical competence, interest in nursing, and engage in nursing in the future were significant predictors of nursing professional commitment. The results of the regression analysis showed that the most significant factor associated with nursing professional commitment was engagement in nursing in the future (*β* = 0.48, *p* < 0.01), followed by clinical competence (*β* = 0.20, *p* < 0.01), interest in nursing (*β* = 0.19, *p* < 0.01) and avoidance behaviour (*β* = −0.13, *p* < 0.05) (Table 4).

**TABLE 4:** Regression analysis of predictors of nursing professional commitment.

Variables	Unstandardized coefficient		Standardised coefficient	*t*	*p*	95% CI		Collinearity statistics	
	*B*	SE	*β*			Lower	Upper	Tolerance	VIF
Constant	0.82	0.21		3.93	< 0.001				
Perceived stress	0.03	0.05	0.03	0.58	0.563	−0.07	0.12	0.50	1.99
Avoidance behaviour	−0.09	0.04	−0.13	−2.47	0.014	−0.16	−0.02	0.51	1.95
Problem‐solving behaviour	0.04	0.03	0.05	1.22	0.222	−0.02	0.10	0.75	1.34
Stay optimistic behaviour	0.06	0.03	0.09	1.84	0.066	−0.00	0.12	0.57	1.76
Transference behaviour	−0.00	0.02	−0.00	−0.07	0.944	−0.05	0.05	0.87	1.16
Clinical competence	0.18	0.04	0.20	4.44	< 0.001	0.10	0.26	0.68	1.48
Interest in nursing	0.11	0.02	0.19	5.05	< 0.001	0.07	0.15	0.90	1.11
Engage in nursing in the future	0.18	0.02	0.48	12.37	< 0.001	0.15	0.21	0.87	1.16

Abbreviations: CI, confidence interval; SE, standard error; VIF, variance inflation factor.

## 4 Discussion

This study aims to explore the level of nursing professional commitment and its influencing factors among graduating nursing students who had completed all clinical practicums, with a special focus on factors related to clinical practice experience and nursing professional commitment. To the best of our knowledge, this is the first study in Taiwan to explore graduating nursing students' professional commitment and influencing factors after experiencing one‐year clinical practicum courses.

The findings of this study indicated that the professional commitment of graduating nursing students was at a moderate level (mean = 2.92 on a 4‐point scale). This result is consistent with recent studies conducted in China, which have likewise reported moderate levels of commitment, including Hua et al. (2022) (2.92/4), Zhao et al. (2022) (2.79/4), and Zhang et al. (2023) (3.26/5). A multidimensional assessment of graduating nursing students' professional commitment is imperative to understand their exact intentions more accurately and deliver targeted interventions for their support. After comparing the scores of the four dimensions, the highest was the score of ‘desire to stay in the profession’, while that of ‘willingness to make an effort’ was the lowest, which was consistent with the study of Zhao et al. (2022). This finding may be interpreted that high employment rates and low risk of unemployment enhance students' willingness to engage in the nursing profession. Nevertheless, these advantages may also lead to a sense of safety and complacency, resulting in a reluctance to invest significant effort in their responsibilities. The goal of nursing education is to ensure graduates not only acquire the essential competence for providing safe and effective patient care but also establish professional commitment to exhibit consistent dedication to nursing. Therefore, increasing students' intrinsic positive value for nursing work and, in turn, increasing their willingness to make efforts in the nursing profession is a top priority in nursing education, so that nursing students can smoothly go through the transition period and reduce their turnover rate when they become new employees after graduation.

This study found that willingness to engage in nursing in the future positively influenced the professional commitment of graduating nursing students. According to our results, students' professional commitment was affected by multiple factors, among which future engagement in nursing emerged as the strongest predictor. This finding aligns with career decision‐making frameworks, such as the SCCT, which posits that individuals' career‐related decisions are shaped by self‐efficacy, outcome expectations, and career interests (Lent and Brown 2019; Lent et al. 1994). In this context, students with a stronger intention to continue in the nursing profession are likely to perceive themselves as more capable and motivated, thereby exhibiting higher levels of professional commitment. Our results are similar to those reported by Nie et al. (2021), which indicated that some nursing students exhibited a low level of professional identity and tended to leave the profession after graduation. The professional commitment of nursing students reflects their loyalty and positive attitude toward the nursing profession, fostering greater engagement in their field (Ying et al. 2023). Our study results showed that only 60.5% of graduating nursing students were determined to work in nursing in the future, which was lower than in previous studies. In the study by Ayaz‐Alkaya and Simones (2022), 99.4% of United States students wished to work as a nurse after graduation and 84.4% of Turkish students wished to work as a nurse after graduation. The shortage of the nursing workforce is a global issue. Since nursing students are the main reserve force of future nursing staff, their willingness to engage in nursing work is vital for alleviating the nursing workforce shortage and promoting high‐quality nursing services. Professional commitment is essential for ensuring nursing stability and optimising resource allocation (Cheng et al. 2021). The goal of nursing education is to encourage students to engage in nursing work after graduation. However, our research results showed that only 60.5% of graduating nursing students were determined to engage in nursing work in the future. To serve as a reference for future coaching and teaching strategies, the reasons why nursing students are unwilling to engage in nursing work after graduation are worthy of in‐depth exploration.

This study found that interest in nursing may positively impact professional commitment of graduating nursing students. This may be because students who are interested in nursing have some understanding of the profession and consider nursing to be an appropriate choice, thus prompting them to consider that they are suited to the nursing profession. This lack of doubt can increase their professional commitment. These results are consistent with those by Zhao et al. (2022). Students may be interested in nursing and therefore would have voluntarily selected nursing, but our results showed that only 58.5% of students were interested in nursing, which is lower than previous studies. In a study by Zhao et al. (2022), 73.0% of the participants were interested in nursing, and 73.3% of those in a study by Ayaz‐Alkaya et al. (2018) were pleased to study nursing. Interest is a key driver of the intention to invest effort in both studying and working (Hua et al. 2022). Students who demonstrate a strong commitment to their profession during their studies are more likely to maintain that commitment after graduation. However, if they lack interest in nursing or disagree with the nursing role, their willingness to engage in a nursing career in the future may be diminished (Chang et al. 2024). Therefore, it is important to promote students' interest in nursing. Nursing educators should incorporate professional values into the curricula to enhance nursing students' beliefs in their professional roles and foster self‐confidence (Zhang et al. 2023).

This study found that clinical competence may positively impact graduating nursing students' professional commitment. Clinical practicums play a vital role in helping nursing students to achieve clinical competence. Therefore, nursing schools arrange various clinical practicum courses for them, which help them gain practical experience in patient care and gradually attain a sense of belonging to the nursing profession while improving their confidence, thereby increasing their professional commitment (Wu et al. 2021). Visiers‐Jiménez et al. (2021) found that graduating nursing students' positive perceptions of their clinical learning environment were associated with a better level of self‐assessed competence. Zhang et al. (2023) showed that students who were dissatisfied with their clinical experience and chose the nursing profession involuntarily were the main factors affecting low‐ and medium‐level nursing professional commitment. This study also found that the students' self‐assessed overall clinical competence was 3.83 among 5, which was moderate. Our results are similar to those of Visiers‐Jiménez et al. (2021), which showed that graduating nursing students self‐assessed their mean overall clinical competence on the VAS as 64.50. Therefore, it is important to further understand the factors that influence the clinical competence of nursing students to serve as a reference for teaching strategies for clinical teachers or preceptors.

This study found that avoidance behaviour may have a negative effect on professional commitment among graduating nursing students. This finding may be interpreted as the lower levels of professional commitment among nursing students, who used avoidance coping strategies more frequently during clinical practicum. Our results are similar to those reported by Ma et al. (2022), which showed that a passive coping style was a significant factor leading to the intention to quit nursing education before graduation. Our study results also found that nursing students mostly use problem‐solving and stay optimistic when facing stress during practicum. However, our results also showed that perceived stress and coping strategies (including problem‐solving behaviour, staying optimistic behaviour, and transference behaviour) during practicum were not predictors of professional commitment of graduating nursing students. Avoidance behaviours exhibited by nursing students during clinical practicum may reflect maladaptive coping responses to clinical stress. Therefore, clinical instructors and preceptors should further understand the underlying reasons for students' avoidance behaviours during practicum, as a basis for adjusting teaching strategies and optimising clinical instruction. In addition, these findings have important implications for curriculum development. Nursing education programs should consider incorporating emotional intelligence and adaptive coping skills into classroom‐based teaching to better prepare students for the demands of clinical practice.

### 4.1 Limitations

This study had some limitations. First, the cross‐sectional design was insufficient to draw conclusions regarding the causality of the associations. A longitudinal study is thus needed to confirm these assertions. Second, this study used a self‐report method to collect data that may not reflect the actual situation. Third, the convenience sampling method used in this study may have affected the generalizability of our results; therefore, caution should be exercised when interpreting our findings. Fourth, although the content validity of the self‐developed Clinical Competence Scale was established via a panel of experts, its construct validity remains to be formally tested. To enhance the scale's psychometric robustness, future research should employ larger sample sizes for confirmatory factor analysis or other construct validation methods. Finally, given the complexity of clinical practicum, there may be other important factors related to nursing professional commitment that were not included in this study. Future research should include more factors related to the clinical practicum, such as teaching attitudes and characteristics of clinical teachers or preceptors.

## 5 Conclusions

This study used a cross‐sectional survey method to analyse professional commitment and influencing factors of graduating nursing students in Taiwan. The results indicated that the professional commitment of graduating nursing students was moderate and that engaging in nursing in the future, clinical competence, interest in nursing, and avoidance behaviour during practicum were the main factors affecting their professional commitment among graduating nursing students. Therefore, nursing educators must comprehensively consider and understand the various factors influencing nursing students' professional commitment and implement targeted interventions. Key recommendations include competency‐based instructional designs and the integration of professional values and emotional intelligence into the curriculum to strengthen students' core competencies, enhance adaptive coping skills, and reinforce beliefs in the professional role. Furthermore, clinical instructors and preceptors should understand the underlying reasons for students' avoidance behaviours during practicum in order to refine teaching strategies and optimise clinical instruction. Through the implementation of these measures, students' professional commitment and willingness to enter the nursing workforce can be effectively enhanced, which is critical for alleviating nursing workforce shortages and ensuring the delivery of high‐quality healthcare services.

## Author Contributions

L.‐H.T.: Study conception and design, Data collection, Data analysis and interpretation, Drafting of the article, Critical revision of the article. J.‐Y.F.: Study conception and design, Critical revision of the article. S.‐F.C.: Data analysis and interpretation.

## Funding

We are grateful to the Ministry of Science and Technology, Taiwan [MOST 110‐2511‐H‐255‐004‐MY2] for their grant support of this study.

## Ethics Statement

We obtained approval from the Institute Review Board, Chang Gung Medical Foundation (201901913B0).

## Conflicts of Interest

The authors declare no conflicts of interest.

## Data Availability

The datasets used and/or analysed during the current study are available from the corresponding author upon reasonable request.
